# 2468. The Genomic Epidemiology of Carbapenemase-Producing Enterobacterales (CPE) in Ontario, Canada, 2016

**DOI:** 10.1093/ofid/ofad500.2086

**Published:** 2023-11-27

**Authors:** Alainna J Jamal, Nathalie Tijet, Allison McGeer, Mahin Baqi, Sergio Borgia, William Ciccotelli, Nataly Farshait, Kevin Katz, Mamta Mehta, Lee Goneau, Dominik Mertz, Lorne N Small, Roberto Melano

**Affiliations:** University of Toronto, Toronto, Ontario, Canada; Public Health Ontario Laboratory, Toronto, Ontario, Canada; Mt. Sinai Hospital, Toronto, Ontario, Canada; William Osler Health System, Toront, Ontario, Canada; William Osler Health System, Toront, Ontario, Canada; Grand River Hospital, Toronto, Ontario, Canada; Humber River Regional Hospital, Toronto, Ontario, Canada; North York General Hospital, Toronto, Ontario, Canada; Grand River Hospital, Toronto, Ontario, Canada; Dynacare, Toronto, Ontario, Canada; Hamilton Health Sciences, McMaster University, Hamilton, ON, Canada; Trillium Health Partners, Toronto, Ontario, Canada; Pam American Health Organization, Toronto, Ontario, Canada

## Abstract

**Background:**

Carbapenemase-producing Enterobacterales (CPE) are among the most urgent of antimicrobial resistance threats. In south-central Ontario, Canada, over one-third of CPE cases are associated with local healthcare and the incidence of such CPE cases is rising steadily. We integrated whole-genome sequencing (WGS) and CPE population-based surveillance data to gain insights into CPE transmission dynamics in Ontario, Canada.

**Figure:**
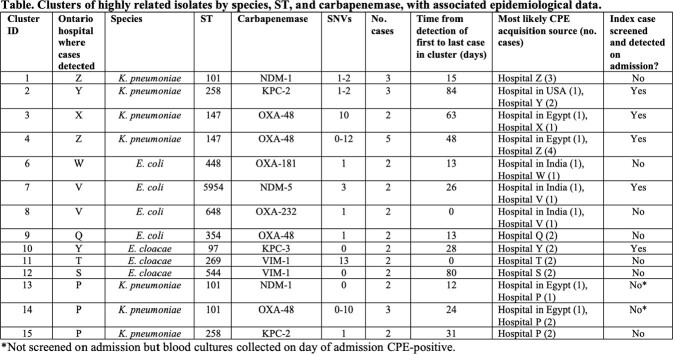

**Methods:**

We included all incident CPE isolates received at the Public Health Ontario Laboratory as part of a voluntary surveillance program from Jan. 1 to Dec. 31, 2016 in Ontario, Canada (population ∼13.5 million). All isolates underwent Illumina WGS. MLST was determined for all isolates, and single nucleotide variant (SNV) analysis was performed by species. SNV analyses were combined with epidemiological data from the Toronto Invasive Bacterial Diseases Network to identify transmission clusters.

**Results:**

There were 206 incident CPE isolates from 176 patients. Most common species were *Escherichia coli* (95, 46%) and *Klebsiella pneumoniae* (76, 37%), and most common carbapenemases produced were NDM (77, 37%), OXA-48-like (68, 33%), and KPC (45, 22%). There was variability in MLST, with ST167 being most common among *E. coli* (10, 11%), and ST147 being most common among *K. pneumoniae* (13, 17%). There were 14 clusters with 34 (17%) CPE isolates belonging to 33 (20%) unique patients total (Table). Cluster size range was 2-5 patients. Time from first to last identified case in clusters ranged from 0 to 258 days. In 8 clusters, the index case likely acquired CPE during prior hospitalization abroad, with subsequent direct or indirect transmission to other patients in the cluster. In 5 clusters, all patients likely acquired CPE at the Ontario hospital where their CPE was detected.

**Conclusion:**

There was variability in CPE species and MLST as well as carbapenemase produced. Almost one fifth of patients belonged to a transmission cluster, with transmission lasting many months in some clusters. These data highlight challenges with CPE local transmission and a need to intensify control measures.

**Disclosures:**

**All Authors**: No reported disclosures

